# Chronic Unexpected Mild Stress Destroys Synaptic Plasticity of Neurons through a Glutamate Transporter, GLT-1, of Astrocytes in the Ischemic Stroke Rat

**DOI:** 10.1155/2019/1615925

**Published:** 2019-03-25

**Authors:** Dafan Yu, Zhenxing Cheng, Abdoulaye Idriss Ali, Jiamin Wang, Kai Le, Enkhmurun Chibaatar, Yijing Guo

**Affiliations:** ^1^Department of Neurology, Affiliated Zhongda Hospital of Southeast University, Nanjing, Jiangsu Province 210009, China; ^2^School of Medicine, Southeast University, Nanjing, Jiangsu Province 210009, China

## Abstract

**Background and Objective:**

Chronic unexpected mild stress (CUMS) destroys synaptic plasticity of hippocampal regenerated neurons that may be involved in the occurrence of poststroke depression. Astrocytes uptake glutamate at the synapse and provide metabolic support for neighboring neurons. Currently, we aim to investigate whether CUMS inhibits synaptic formation of regenerated neurons through a glutamate transporter, GLT-1, of astrocytes in the ischemic stroke rats.

**Method:**

We exposed the ischemic stroke rats to ceftriaxone, during the CUMS intervention period to determine the effects of GLT-1 on glutamate circulation by immunofluorescence and mass spectrometry and its influences to synaptic plasticity by western blot and transmission electron microscopy.

**Result:**

CUMS evidently reduced the level of astroglial GLT-1 in the hippocampus of the ischemic rats (*p* < 0.05), resulting in smaller amount of glutamate being transported into astrocytes surrounding synapses (*p* < 0.05), and then expression of synaptophysin was suppressed (*p* < 0.05) in hippocampal dentate gyrus. The ultrastructures of synapses in dentate gyrus were adversely influenced including decreased proportion of smile synapses, shortened thickness of postsynaptic density, reduced number of vesicles, and widened average distance of the synaptic cleft (all *p* < 0.05). Moreover, ceftriaxone can promote glutamate circulation and synaptic plasticity (all *p* < 0.05) by raising astroglial GLT-1 (*p* < 0.05) and then improve depressive behaviors of the CUMS-induced model rats (*p* < 0.05).

**Conclusion:**

Our study shows that CUMS destroys synaptic plasticity of regenerated neurons in the hippocampus through a glutamate transporter, GLT-1, of astrocytes in the ischemic stroke rats. This may indicate one potential pathogenesis of poststroke depression.

## 1. Introduction

Stroke is one of the diseases with the highest fatality rate in the elderly population worldwide, with incidence rates between 10 and 200 per 10000 individuals in the age range of 55-85 [1]. Unfortunately, depression occurs frequently in patients after stroke, with the poor functional outcome [2] and high mortality [3]. 27.47% of stroke patients suffer from depression 2 weeks later [4], so poststroke depression (PSD) is of high clinical importance [1]. The pathogenesis of PSD includes disorders in neural plasticity and in glutamate neurotransmission [5], ascending monoamine systems [6], and so on. However, the precise pathological mechanism of poststroke depression is not yet clear.

It is well known that glutamate is the main excitatory neurotransmitter in the mammalian central nervous system, which executes its functions by activating ionotropic and metabotropic glutamate receptors [7]. In stroke, overreleased glutamate accumulates in the synaptic cleft and overactivates metabotropic glutamate receptor (mGluR) inducing the death of neurons [8]. The process is known as “excitotoxicity” [9] which is associated with depression [10]. Astroglial glutamate transporter-1 (GLT-1) in rodents, also known as excitatory amino acid transporter-2 (EAAT-2) in human, functions to clear 90% glutamate spilled over the synaptic cleft [11, 12]. In physiology, glutamate transported into astrocytes is converted into glutamine by glutamate dehydrogenase (GS), which is released to extracellular and then absorbed by neurons. And then, glutaminase (GLS) converts glutamine into L-glutamate and ammonia in neurons [13].

In the central nervous system, astrocytes not only protect neurons by maintaining the stability of microenvironment [14] but also participate in the formation of synaptic networks [15] and modulation of synaptic transmission [16]. Our prior study found that increased newborn astrocytes in the hippocampus were closely related with decreased exploration ability and anhedonia of the model rats, accompanied with decreased newborn neurons and synaptic density [17, 18]. Hippocampal dentate gyrus (DG) is closely related to the memory and exploration ability [5]. On one hand, patients with cerebral infarction on the left brain are more likely to have depression, and hippocampal volume of depressed patients is significantly smaller. On the other hand, chronic unexpected mild stress (CUMS) induces hippocampal astrocytes proliferating after stroke [17] while the synaptogenesis is negatively affected [19]. Our recent study also has found that newborn astrocytes of the model rats do not express sufficient quantities of GLT-1 in vitro [20]. These beg a question whether CUMS influences synaptic plasticity of hippocampal DG through a glutamate transporter, GLT-1, of astrocytes in the ischemic stroke rat.

Based on the well-recognized rat model which combines middle cerebral artery occlusion (MCAO) with CUMS [21], we investigated the precise relationship among GLT-1, glutamate circulation, synaptic plasticity, and depressive behaviors by treating rat models of PSD with a beta-lactam antibiotic ceftriaxone (Cef) that increases GLT-1 expression [22].

## 2. Methods and Materials

### 2.1. Animals

Adult male Sprague-Dawley (SD) rats (230-260 g) were supplied by Medical School of Southeast University, Nanjing, China. The rats were housed in a temperature- and humidity-controlled environment. After being housed for 1 week, the rats' excess weights were about 20 g and then they were assigned to different groups for experimental intervention. All experimental procedures and animal study were executed with the consent of the National Institutes of Health Guide for the Care and Use of Laboratory Animals. This experiment was also approved by the Animal Ethical and Welfare Committee of Southeast University.

### 2.2. Middle Cerebral Artery Occlusion (MCAO) Model

Permanent focal cerebral ischemia was induced by intraluminal middle cerebral artery occlusion [23]. Adult male SD rats (250-280 g) were anaesthetized with sodium pentobarbital (40 mg/kg) by intraperitoneal injection (i.p.). A surgical midline incision was made to expose and isolate the left CCA, external carotid arteries (ECA), and internal carotid arteries (ICA). A small incision was made in the CCA nearby its bifurcation, and a 3/0 gauge monofilament nylon suture coated in poly-L-lysine was inserted up to the origin of the MCA through ICA. The length of the suture in CCA and ICA was approximately 18.5 mm. Rats in the control group were treated with the same surgical procedures until making incision in the CCA. One day later, neurological evaluation was conducted [24]. The rats scaled 0, 3, and 4 were excluded from this study.

### 2.3. Chronic Unpredictable Mild Stress (CUMS) Model

The chronic unpredictable mild stress model was conducted [25] with minor modifications. Nine different stressors were arranged to occur day and night in a random order for 18 consecutive days as follows: 45° cage tilt for 22 h, food and water deprivation for 20 h, and water deprivation for 21 h, followed by 1 h exposure to empty bottles, 23 h period of soiled cage, overnight illumination (lights on for a total of 36 h), 2 h period of caging, tail pinch (1 min), foot shock (5 s every minute for 30 min, voltage in intensity was adjusted enough to elicit jumping or squeaking), and swimming in 4°C water (5 min). The rats were housed in separate cages and had no contact with the other stressed rats.

### 2.4. Drug Administration

**Figure 1:**
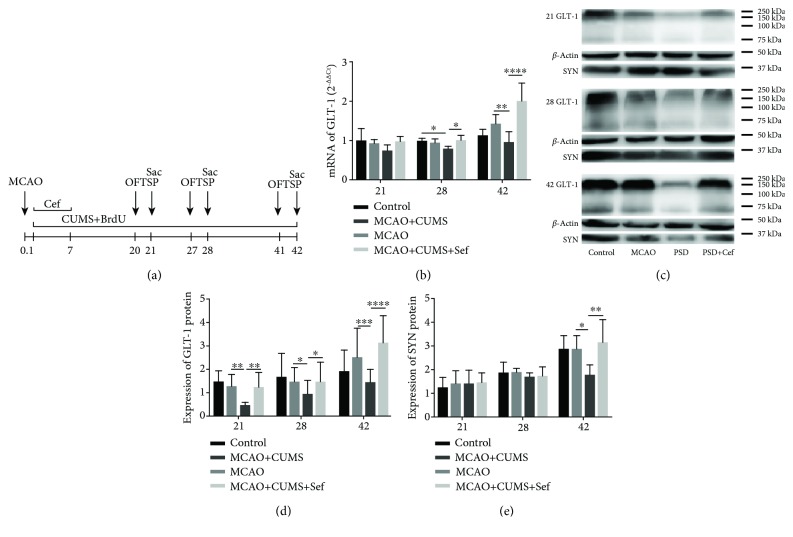
(a) Schematic of the experimental designs. (b) Ceftriaxone reversed the decrease of GLT-1 mRNA in the hippocampus of the CUMS-induced model rats on the 28th day and the 42nd day. (c) Representative western blotting pictures showed proteins of GLT-1 and synaptophysin (SYP, SYN) in the hippocampus on the 21st/28th/42nd day. (d) Ceftriaxone reversed the decrease of GLT-1 protein in the hippocampus of the CUMS-induced model rats at the three time points. (e) SYN decreased on the 42nd day and then was reversed by ceftriaxone. ^∗^*p* < 0.05, ^∗∗^*p* < 0.005, ^∗∗∗^*p* < 0.0005, ^∗∗∗∗^*p* < 0.0001, one-way ANOVA (Tukey), *n* = 6.

Ceftriaxone (Cef) was purchased from Roche Biotechnology (Basel, Switzerland). 5-Bromo-2′-deoxyuridine (BrdU) was purchased from Sigma (USA). One hundred and eight adult male SD rats were randomly assigned into four groups: (1) control group, (2) MCAO group, (3) MCAO+CUMS group, and (4) MCAO+CUMS+Cef group, with rats treated with 200 mg/kg Cef (Roche Biotechnology, Basel, Switzerland) in 1.0 ml 0.9% saline (intraperitoneal injection, i.p.) once a day for 7 days after surgery (Table 1 and Figure 1(a)). At the same time, rats in each subgroup received 50 mg/kg 5-bromo-2′-deoxyuridine [26] (BrdU, Sigma, USA) in 1.0 ml 0.9% saline (i.p.) once a day until being sacrificed (Sac). On the 20^th^, 27^th^, and 41^st^ days, rats were evaluated for general locomotor and rearing activity by the open-field test (OFT). On the 21^st^, 28^th^, and 42^nd^ days, the sucrose preference (SP) test was used to operationally determine whether the rats had anhedonia [21]. After the sucrose preference test, rats were sacrificed in each subgroup.

### 2.5. qRT-PCR Analysis

The level of GLT-1 mRNA was measured on the 21^st^, 28^th^, and 42^nd^ days. Total RNA of left cerebral hippocampal DG was extracted by Trizol (Thermo Scientific, USA) according to the manufacturer's instructions. The RNA was reverse transcribed into cDNA using the RevertAid first strand cDNA synthesis kit (Thermo Scientific, USA). Amplify the cDNA with SYBR® green PCR master mix (Thermo Scientic, USA), and fluorometric quantify it by StepOne™ Software 2.3 (Thermo Scientific, USA). The primer sequences were designed as follows: GLT-1 (94 bp), forward primer 5′-GTGGACTGGCTGCTGGATAG-3′, reverse primer 5′-GCTCGGACTTGGAAAGGTGA-3′; and GAPDH (452 bp), forward primer 5′-ACCACAGTCCATGCCATCAC-3′, reverse primer 5′-TCCACCACCCTGTTGCTGTA-3′. The levels of subgroups were calculated as 2^-ΔΔCt^.

### 2.6. Western Blotting Analysis

The levels of GLT-1 and synaptophysin proteins were measured on the 21^st^, 28^th^, and 42^nd^ days. Total protein of left cerebral hippocampal DG was extracted by a protein extraction kit (Vazyme Biotechnology, China) according to the manufacturer's instructions. Their concentrations were determined by a BCA protein assay reagent kit (KeyGen Biotech, China). Using SDS-PAGE, the equal amounts of protein were separated from different subgroups. Then, they were transferred to NC membranes. After blocking with 5% nonfat milk for one hour at 37°C, primary antibodies rabbit anti-GLT-1 (1 : 1000, Abcam, UK), anti-synaptophysin (1 : 1000, Abcam, UK), and anti-*β*-actin [27] (1 : 1000, CST, USA) were added overnight at 4°C and washed. The secondary antibody HRP Affinipure goat anti-rabbit IgG (H+L) (1 : 2000, FCMACS, China) was used to incubate the membranes for one hour at 37°C. Images were analyzed with ImageJ (National Institutes of Health, USA).

### 2.7. Immunofluorescence, Cell Count, and Fluorescence Intensity Determination

**Figure 2:**
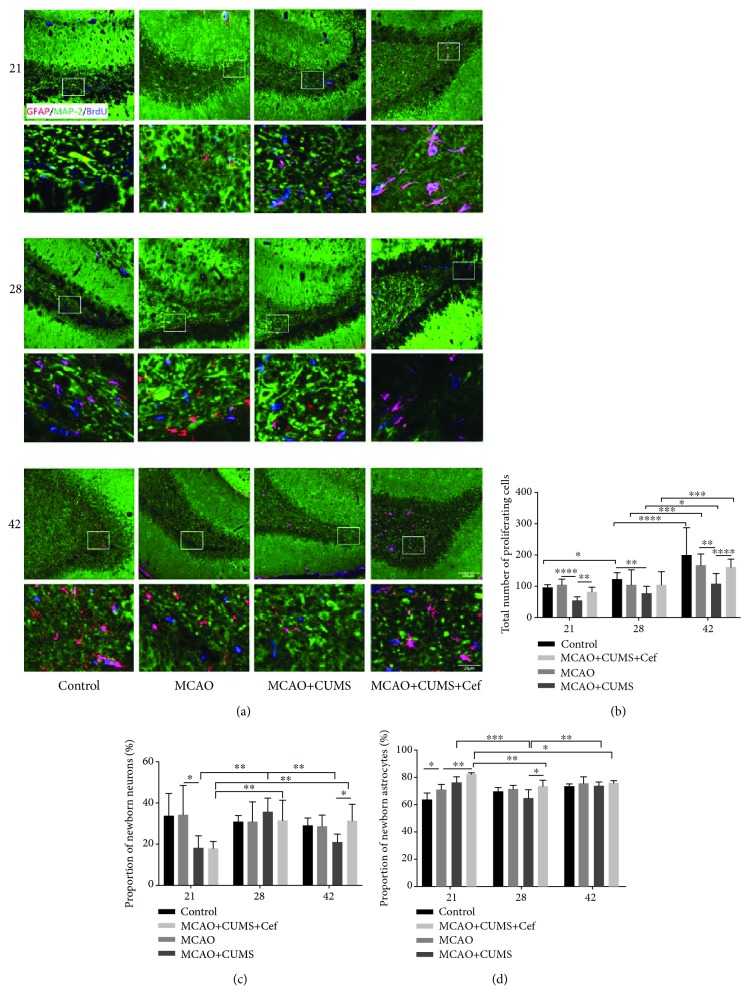
Ceftriaxone improved regeneration capacity impaired in the hippocampal DG of the CUMS-induced model rats. (a) Representative immunofluorescent photomicrographs (200×) of GFAP/MAP2/BrdU (red/green/blue) in hippocampal DG were pictured on the 21st/28th/42nd day. Scale bars: 100 *μ*m and 20 *μ*m. (b) Ceftriaxone promoted the proliferation of total newborn cells in hippocampal DG of the CUMS-induced model rats. The newborn nuclei were labeled by BrdU (blue). (c) Ceftriaxone influenced the proportion of newborn neurons (MAP2/BrdU, green/blue) in the total newborn cells. (d) Ceftriaxone raised the proportion of newborn astrocytes (GFAP/BrdU, red/blue) in the total newborn cells. ^∗^*p* < 0.05, ^∗∗^*p* < 0.005, ^∗∗∗^*p* < 0.0005, ^∗∗∗∗^*p* < 0.0001, two-way ANOVA (Tukey), *n* = 6.

Animals were perfused with 4% PFA before brain harvesting. After 30% sucrose dehydration, the brains were fixed in 4% paraformaldehyde for frozen section. Coronal brain slices of 20 *μ*m were fixed with 4% paraformaldehyde for 30 min at 37°C and washed three times with PBS. Then, antigen was repaired with 0.4% pepsin for 10 min at 37°C and incubated with 2 mmol HCL for 30 min at 37°C. The sections were washed three times with PBS and treated with 0.3% Triton X-100 and 1% BSA for 30 min at 37°C. Subsequently, sections were incubated overnight at 4°C with the following primary antibodies: anti-BrdU antibody (1 : 100, Abcam, UK), anti-GFAP antibody (1 : 100, Abcam, UK), anti-NeuN antibody (1 : 100, Abcam, UK), anti-L-glutamate antibody (1 : 100, Abcam, UK), anti-glutamine antibody (1 : 100, Abcam, UK), and anti-MAP2 antibody (1 : 100, CST, USA). After washing three times, sections were incubated for 1 h with Alexa Fluor 405 AffiniPure goat anti-rat IgG (1 : 100, Abcam, UK), Alexa Fluor 647 AffiniPure goat anti-mouse IgG (1 : 100, FCMACS, China), and Alexa Fluor 488 AffiniPure goat anti-rabbit IgG (1 : 100, FCMACS, China). A laser confocal microscope (Olympus, FV1000, Japan) was used to quantify immunopositive cells and fluorescence intensity in sections. At least five noncontinuous sections per rat were selected and photographed at DG (200×). The noncontinuous sections were apart with each other about 100 *μ*m. All the sections were taken from the same brain region DG across animals and groups. The positive numbers of BrdU, NeuN/BrdU, and GFAP/BrdU cells in different cells were counted and expressed as percentages as shown in Figure 2. The fluorescence intensity of Glu and Gln in Glu/NeuN/BrdU, Gln/NeuN/BrdU, Glu/GFAP/BrdU, and Gln/GFAP/BrdU cells was determined. The HV of Alexa Fluor 488 in CHS2 (Glu and Gln) was settled at 795 and kept constant for all slices.

### 2.8. Transmission Electron Microscopy (TEM) and Measure

1 mm^3^ of DGs was fixed in 2.5% glutaraldehyde in at 4°C. Tissues were sliced by vibratome and stained by lead. At least ten sections per rat were selected and photographed in DG (40000×) by a transmission electron microscope (Hitachi H-7650, Japan). Synapses were measured by Image-Pro Plus 6.0 (Media Cybernetics, USA). The thickness of the postsynaptic density (PSD) and the average distance between presynaptic membrane and postsynaptic membrane were measured [28, 29].

### 2.9. Mass Spectrometry

Rats' cerebrospinal fluid (CSF) was collected for multiple reaction monitoring (MRM). The internal standard working liquid of glutamate and glutamine was 1 : 1 mixed with 10% sulfosalicylic acid solution, and then they were 1 : 5 mixed with 5% salicylic acid. 140 *μ*l mixed internal standard precipitation liquid was added in the sample, the standard area, and the blank spot. The blank matrix was pure water, and the double blank was not included in the internal standard. The samples were mixed, centrifuged, diluted twice, and then tested.

### 2.10. Statistical Analysis

Data were plotted in PRISM 7.0 or SPSS and expressed as the mean ± SEM. One-way analysis of variance (ANOVA) or two-way ANOVA (Tukey or Newman-Keuls) was used for statistical analysis. *p* values < 0.05 were considered statistically significant.

## 3. Results

### 3.1. CUMS Inhibited the Expression of GLT-1 in the Hippocampus of the Ischemic Stroke Rats

We firstly investigated the level of GLT-1 in the hippocampus of the CUMS-induced model rats. In the infarcted (left) side, the level of GLT-1 mRNA was inhibited by CUMS on the 28th day (*n* = 6, *p* = 0.0146) and 42nd day (*n* = 6, *p* = 0.0045, Figure 1(b)). At the translation level, GLT-1 protein was also decreased on the 21st, 28th, and 42nd days (*p* < 0.05, Figures 1(c) and 1(d)). Treatment of ceftriaxone gradually promoted the increase of GLT-1 both in transcription and translation levels from the 21st day to the 42nd day (*n* = 6, *p* < 0.05).

### 3.2. CUMS Impaired Regeneration Capacity in the Hippocampal DG of the Ischemic Stroke Rats

As time went on, the total number of proliferating cells increased, but the number of proliferating cells in hippocampal DG of the CUMS-induced model rats was significantly lower than that in MCAO rats (*n* = 6, *p* < 0.05, Figures 2(a) and 2(b)). It was noteworthy that ceftriaxone promoted the proliferation of total newborn cells in hippocampal DG of the CUMS-induced model rats by upregulating astroglial GLT-1 (*p* < 0.05). At the 21st day, the proportions of newborn neurons in the CUMS-induced model rats and ceftriaxone-treated rats were 18.47% and 17.79%, which were lower than those in the control (33.62%) and MCAO rats (34.19%) (*p* < 0.005, Figure 2(c)). Seven days later, the proportion of newborn neurons in the CUMS-induced model rats (35.55%) was higher than the other groups, although ceftriaxone also increased the percentage to 31.31%. 14 days later, the proportion in the CUMS-induced model rats fell back to 21.09% and in the Cef-treated rats was 30.73% which is close to the proportion in the control and MCAO rats.

On the 21st day, the proportion of newborn astrocytes in the CUMS-induced model rats was 76.38%, which was higher than 71.01% in the MCAO rats, and ceftriaxone increased the proportion in the CUMS-induced model rats to 82.24% (*p* < 0.05, Figure 2(d)). On the 28th day, the proportion of newborn astrocytes in the CUMS-induced model rats reduced to 64.44%, which was lower than that in the ceftriaxone-treated rats (72.98%) (*p* = 0.0184). On the other hand, volume and arborisation of them in the CUMS-induced model rats increased accompanied with upregulated GLT-1 (Figure 2(a)). Combining with the above data, we found that the changes of newborn astrocytes induced by ceftriaxone in the hippocampus were earlier than newborn neurons.

### 3.3. CUMS Restrained the Circulation of Glutamate in Hippocampal DG of the Ischemic Stroke Rats

**Figure 3:**
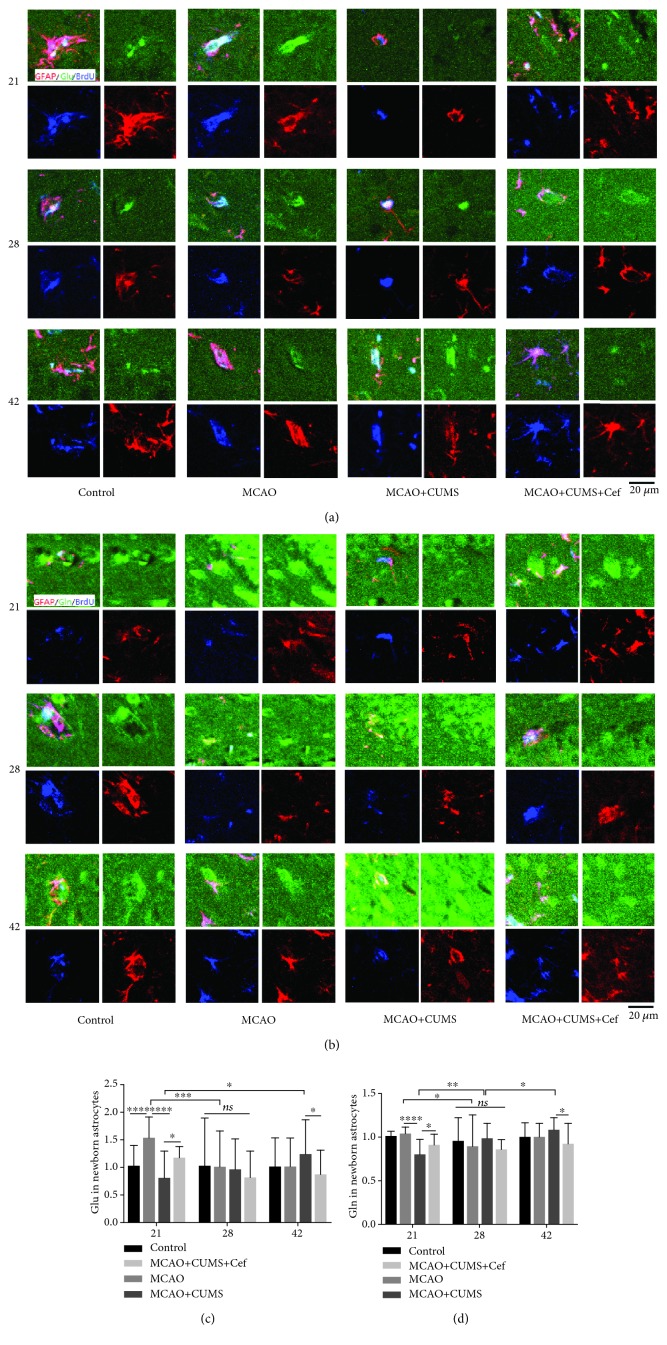
Ceftriaxone promoted newborn astrocytes in hippocampal DG in the CUMS-induced model rats transporting glutamate. (a) Representative immunofluorescent photomicrographs (200×) of glutamate (Glu, green) in newborn astrocytes (GFAP/BrdU, red/blue) were pictured on the 21st/28th/42nd day. Scale bars: 20 *μ*m. (b) Representative immunofluorescent photomicrographs (200×) of glutamine (Gln, green) in newborn astrocytes (GFAP/BrdU, red/blue) on the 21st/28th/42nd day. Scale bars: 20 *μ*m. (c) Fluorescence intensity of glutamate in newborn astrocytes was modulated by astroglial GLT-1. (d) Trend of glutamine intensity was paralleled with glutamate in newborn astrocytes in hippocampal DG. ^∗^*p* < 0.05, ^∗∗^*p* < 0.005, ^∗∗∗^*p* < 0.0005, ^∗∗∗∗^*p* < 0.0001, two-way ANOVA (Tukey), *n* = 6.

In our research, newborn astrocytes in the hippocampal DG of the MCAO rats transported excessive glutamate from the synapse clefts in the acute phase on the 21st day (*n* = 6, *p* = 0.0001) and decreased to normal on the 28th and 42nd days (Figures 3(a) and 3(c)). CUMS reduced the expression of astroglial GLT-1 and impaired the ability of newborn astrocytes in the hippocampal DG transporting excessive glutamate on the 21st day (*n* = 6, *p* < 0.0001). Maybe due to glutamate accumulating in the synapse clefts continuously, glutamate transported by newborn astrocytes on the 42nd day was more than the 21st day (*n* = 6, *p* = 0.0429). However, glutamate in newborn astrocytes in the ceftriaxone-treated rats was more than that in the CUMS-induced model rats on the 21st day (*n* = 6, *p* = 0.0494) and then downed to normal on the 28th and 42nd days. These data suggested that ceftriaxone improved the ability of newborn astrocytes in transporting glutamate by upregulating astroglial GLT-1. Glutamine intensities converted from glutamate in newborn astrocytes in hippocampal DG of the different groups were shown in Figures 3(b) and 3(d). On the 21st day, glutamine in newborn astrocytes in the CUMS-induced model rats was significantly less than that in the MCAO rats (*n* = 6, *p* < 0.0001) and increased in ceftriaxone-treated rats (*n* = 6, *p* = 0.0318). Another 21 days later, glutamine in newborn astrocytes in the CUMS-induced model rats decreased due to the intervention of ceftriaxone (*n* = 6, *p* = 0.0116). Overall, the trend of glutamine was paralleled with glutamate in newborn astrocytes in hippocampal DG.

**Figure 4:**
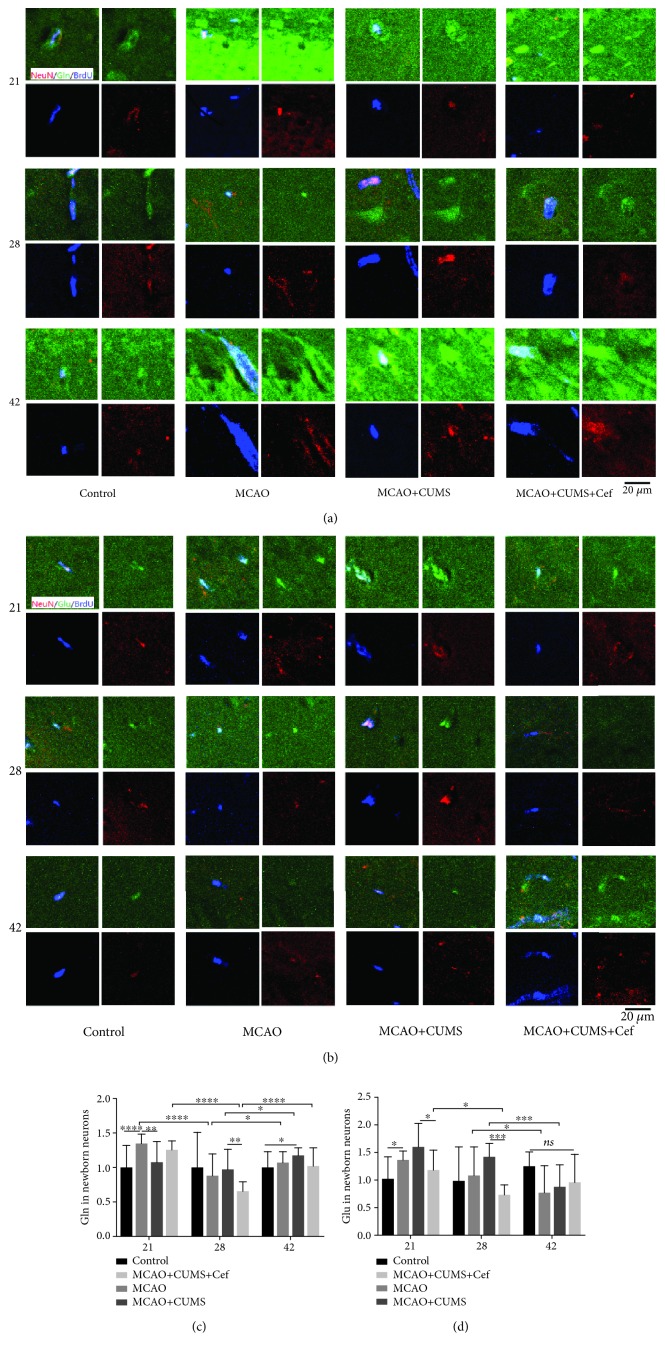
Ceftriaxone alleviated the accumulation of glutamate in newborn neurons in hippocampal DG in the CUMS-induced model rats. (a) Representative immunofluorescent photomicrographs (200×) of glutamine (Gln, green) in newborn neurons (NeuN/BrdU, red/blue) were pictured on the 21st/28th/42nd day. Scale bars: 20 *μ*m. (b) Representative immunofluorescent photomicrographs (200×) of glutamate (Glu, green) in newborn neurons (NeuN/BrdU, red/blue) on the 21st/28th/42nd day. Scale bars: 20 *μ*m. (c) Fluorescence intensity of glutamine in newborn neurons was influenced by the level of astroglial GLT-1. (d) Accumulation of glutamate in newborn neurons in hippocampal DG in the CUMS-induced model rats was alleviated by upregulated astroglial GLT-1. ^∗^*p* < 0.05, ^∗∗^*p* < 0.005, ^∗∗∗^*p* < 0.0005, ^∗∗∗∗^*p* < 0.0001, two-way ANOVA (Tukey), *n* = 6.

To further make the circulation of glutamate in hippocampal DG of the CUMS-induced model rats clear, we measured the intensities of glutamate and glutamine in newborn neurons. We found that more glutamine was uptaken into neurons in the MCAO rats on the 21st day (*n* = 6, *p* < 0.0001), then reduced to normal (Figures 4(a) and 4(c)). Relatively, glutamine in the MCAO+CUMS group was lower than MCAO group on the 21st day, which increased gradually and was higher than the control rats on the 42nd day (*n* = 6, *p* = 0.0444). Upregulated GLT-1 reversed the glutamine intensity in newborn neuron, especially on the 28th day (*n* = 6, *p* = 0.0014). It was worth noting that upregulated astroglial GLT-1 also significantly reduced glutamate intensities in newborn neurons in ceftriaxone-treated rats on the 21st and 28th days (*n* = 6, *p* < 0.05, Figures 4(b) and 4(d)), thereby alleviated the accumulation of glutamate in neurons.

### 3.4. CUMS Induced the Accumulation of Glutamate in Cerebrospinal Fluid of the Ischemic Stroke Rats

**Figure 5:**
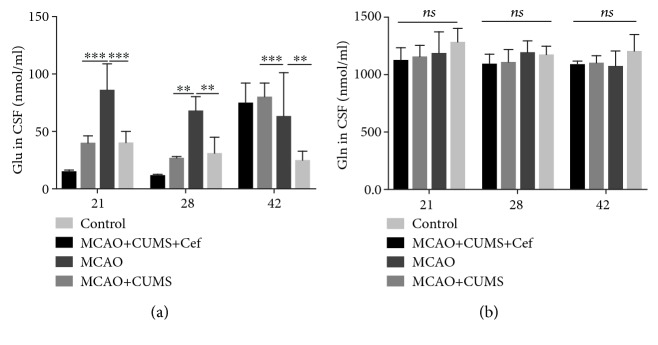
Ceftriaxone reduced the concentration of glutamate in cerebrospinal fluid (CSF) of PSD rats. (a) Ceftriaxone reversed the accumulation of glutamate in CSF of the CUMS-induced model rats measured by MS-MRM on the 21st/28th/42nd day. (b) Concentration of glutamine in CSF of these subgroups had no obvious difference. ^∗∗^*p* < 0.005, ^∗∗∗^*p* < 0.0005, one-way ANOVA (Tukey), *n* = 5.

In order to further evaluate the influence of ceftriaxone on glutamate circulation, we measured the concentrations of glutamate and glutamine in CSF of rats by MS-MRM. The level of glutamate in CSF of the CUMS-induced model rats was significantly higher than that of the MCAO rats at the three time points (*n* = 5, *p* < 0.005, Figure 5(a)). Ceftriaxone reversed the accumulation of glutamate in CSF of the CUMS-induced model rats (*n* = 5, *p* < 0.005). However, glutamine in CSF of these subgroups did not differ with each other (*n* = 5, *p* > 0.05, Figure 5(b)).

### 3.5. CUMS Adversely Affected Synaptic Plasticity in Hippocampal DG of the Ischemic Stroke Rats

**Figure 6:**
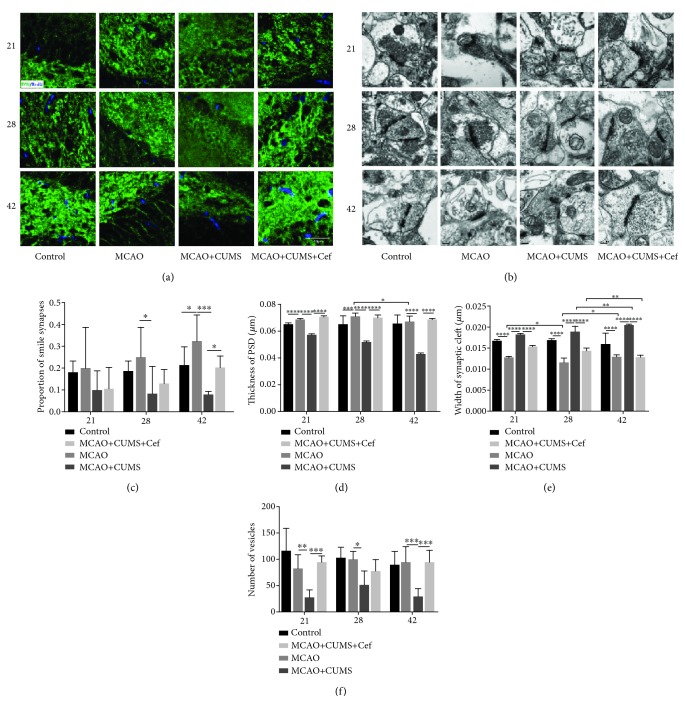
Ceftriaxone improved synaptic plasticity in hippocampal DG of the CUMS-induced model rats. (a) Immunofluorescent images (1000×) of SYN (green) in the hippocampal DG on the 21st/28th/42nd day. Slices were also treated with BrdU to label new nuclei (blue). Scale bar: 40 *μ*m. (b) Representative transmission electron images of synapses in DG (40000×) were pictured at the three time points. Scale bar: 0.2 *μ*m. (c) Proportion of smile synapses of the CUMS-induced model rats was lower especially on the 42nd day. (d) Thickness of PSD in each synapse was smaller in the CUMS-induced model rats. (e) Average width of the synaptic cleft in synapses of the CUMS-induced model rats was significantly wider. (f) Decreased numbers of vesicles in synapses in the CUMS-induced model rats were reversed by ceftriaxone. ^∗^*p* < 0.05, ^∗∗^*p* < 0.005, ^∗∗∗^*p* < 0.0005, ^∗∗∗∗^*p* < 0.0001, two-way ANOVA (Tukey), *n* = 3.

The effects of ceftriaxone on synaptic plasticity were measured at two aspects including quantity and ultrastructure in hippocampal DG of the CUMS-induced model rats. Synaptophysin (SYP, SYN), a protein in synaptic vesicular membrane, decreased on 42nd day (*n* = 6, *p* = 0.0239) and was reversed by the intervention of ceftriaxone (*n* = 6, *p* = 0.0012, Figures 1(c), 1(e), and 6(a)). In terms of synaptic ultrastructure in hippocampal DG, the proportion of smile synapses of the CUMS-induced model rats was lower than that of the MCAO rats especially on the 42nd day (*n* = 3, *p* = 0.0001, Figures 6(b) and 6(c)). Thickness of the postsynaptic density (PSD) in the CUMS-induced model rats was smaller on the three time points (*n* = 3, *p* < 0.05, Figures 6(b) and 6(d)); the average distance between presynaptic membrane and postsynaptic membrane was significantly wider (*n* = 3, *p* < 0.05, Figures 6(b) and 6(e)), and the number of vesicles in DG was less (*n* = 3, *p* < 0.05, Figures 6(b) and 6(f)). After upregulating astroglial GLT-1 by ceftriaxone, smile synaptic proportion increased in the CUMS-induced model rats on the 42nd day (*n* = 3, *p* = 0.0553). PSD, average width of the synaptic clefts, and numbers of vesicles in synapses in the CUMS-induced model rats were also reversed by ceftriaxone (*p* < 0.05). Synaptic quantity and ultrastructure in hippocampal DG suggested that ceftriaxone reversed adverse synaptic plasticity in the CUMS-induced model rats.

### 3.6. Depressive Behaviors of the Ischemic Stroke Rats Induced by CUMS Were Improved by Ceftriaxone

**Figure 7:**
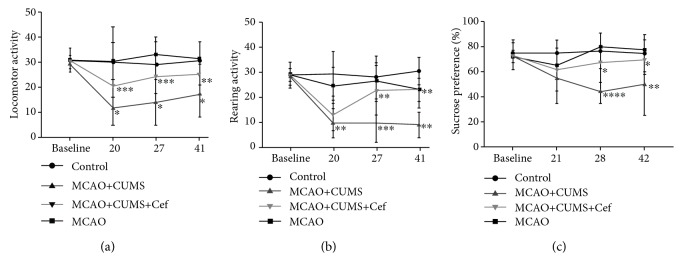
Ceftriaxone improved depressive behaviors of the CUMS-induced model rats. (a) Ceftriaxone improved general locomotor activity of the CUMS-induced model rats on the 27th day. (b) Ceftriaxone improved rearing activity of the CUMS-induced model rats on the 27th and 41st days. (c) Ceftriaxone improved anhedonia of the CUMS-induced model rats on the 28th and 42nd days. ^∗^*p* < 0.05, ^∗∗^*p* < 0.005, ^∗∗∗^*p* < 0.0005, ^∗∗∗∗^*p* < 0.0001, one-way ANOVA (Tukey), *n* = 6.

Open-field test (OFT) evaluates locomotor and rearing activity, and sucrose preference test evaluates the degree of anhedonia. In our study, CUMS caused the decrease of investigative ability of the MCAO rats on the 20th, 27th, and 41st days (*n* = 6, *p* < 0.05, Figures 7(a) and 7(b)). On the 28th and 42nd days, anhedonia also happened in the CUMS-induced model rats (*n* = 6, *p* < 0.05, Figure 7(c)). After ceftriaxone upregulated astroglial GLT-1, depressive behaviors of the CUMS-induced model rats were obviously improved on the 27th/28th and 41st/42nd days. The improvement of depressive behaviors is inseparable from the active synaptic plasticity.

## 4. Discussion

The present study shows that CUMS repressed the transcription and translation of astroglial GLT-1 in hippocampal DG of the MCAO rats, significantly depressed the ability of astrocyte transporting glutamate accompanied with the reduction of regeneration capacity, unbalanced ratio of newborn neurons and astrocytes and adverse synaptic plasticity, and then leaded to depressive behaviors including decreased investigative ability and anhedonia. However, ceftriaxone reversed these pathological changes by upregulating astroglial GLT-1 in hippocampal DG of the CUMS-induced model rats and eventually improved its depressive behaviors.

The previous studies show that ceftriaxone upregulates astroglial GLT-1 within 24 h after pretreatment and then downregulates after one week in brain ischemia, traumatic brain injury, and so on [22, 30, 31]. In contrast, we found that GLT-1 was still high on the 42nd day after the treatment of ceftriaxone for 7 days consecutively accompanied by CUMS. Compared with MCAO models, the effects of ceftriaxone lasted longer in the CUMS-induced model rats, and the underlying mechanism maybe was that the upregulation of GLT-1 initiated by ceftriaxone was maintained by the persistent exclusive glutamate.

GLT-1 is mainly expressed in the cytoplasm and the protuberant ends of astrocytes, partly expressed in neurons [32], including monomers (62 kDa), dimers (120 kDa), and multimers (about 180 kDa). Among them, multimer of GLT-1 is the main active ingredient [33]. Both of the transcription and translation levels are especially important to the function of astroglial GLT-1 [34]. Upregulated GLT-1 can reduce the damage of excitotoxicity in rat models of cerebral ischemia [35]. In our research, CUMS downregulated glial GLT-1 mRNA/protein in the hippocampus, especially multimer of GLT-1, and then disrupted the homeostasis of glutamate in the brain. At the same time, the proportion of newborn astrocytes in the CUMS-induced model rats increased paralleled with narrow volume and arborisation. These results indicated that the newborn astrocytes in the PSD rats were immature functionally and could not maintain the homeostasis of glutamate. A study shows that the upregulation of GLT-1 via ceftriaxone administration has detrimental effects on spatial learning and memory in rats [36]. But in our research, upregulated astroglial GLT-1 improved CUMS-induced model rats' depressive behaviors including exploration ability and anhedonia. Both the two behavioral assessments (OFT and sucrose preference test) were only improved 7 days after the increase of astroglial GLT-1 in the CUMS-induced model rats.

MCAO causes a large release of glutamate in the brain, which induces the neurons to overexcite and damages the brain tissue. This is excitatory toxicity of glutamate [37]. Astroglial GLT-1 functions to clear 90% glutamate spilled over the synaptic cleft [11, 12] and then relieves excitatory toxicity of glutamate. Combined with the decrease of astroglial GLT-1 in the CUMS-induced model rats, we found that glutamate circulation in hippocampal DG of the CUMS-induced model rats was affected. Physiologically, glutamate is transported by astroglial GLT-1 and then converted to glutamine by glutamine synthetase (GS) in astrocytes. Glial glutamine is released into the extracellular space and then uptaken into neurons by SNAT1/2/6/7. The glutamine in neurons is deammoniated to glutamate by glutaminase (GLS). The part of the glutamate in the neuron is secreted by vesicles to the synaptic clefts and then absorbed by another neuron. The part of glutamate in the neuronal cytoplasm is converted to *α*-ketone glutaric acid (*α*-KG) by glutamate dehydrogenase (GDH), which is transferred into the mitochondria for energy provision. The rest of the glutamate is transformed into GABA by GAD67 [13]. Due to low expressed astroglial GLT-1, decreased glutamate in newborn astrocyte of the CUMS-induced model rats significantly affected the metabolism of glutamate in neurons. Glutamate accumulated in the synaptic cleft inhibited synaptic plasticity.

Presynaptic axons and postsynaptic dendrites, along with the perisynaptic astroglia which ensheathe the vast majority of all synapses, constitute the core elements of the central nervous system functional unit and neuron-astrocyte synaptic complex. It is reported that astroglial GLT-1 has positive effect on synaptic formation postnatally [38]. Yang et al. also reported that transcriptional activation of astroglial GLT-1 was related to axonal interactions with astroglia closely [39]. The immature astrocytes with low expressed GLT-1 in the hippocampus of the CUMS-induced model rats blocked the formation and maturation of synapses, including reduced SYN, reduced the number of excitatory synaptic vesicles, widened the synaptic cleft, and reduced the postsynaptic density of excitatory synapses. SYN is a transmembrane glycoprotein found in presynaptic vesicles of the nerve cells [40]. The decrease of SYN in hippocampal DG of the CUMS-induced model rats suggested that the transmission of the neurotransmitter between synapses or the number of synapses reduced. In the ultrastructure of synapses, smile synapse (negative synapse) is newborn synapse and frown synapse (positive synapse) is mature synapse [41]. The reductions of smile synapse and the number of SYN indicated that synapses were decreased by CUMS. On one hand, thickness of the postsynaptic density (PSD) is associated with synaptic excitability [29]. Synapses with long PSD are excitatory synapses, while synapses with short PSD are inhibitory synapses mostly. There are a large number of synaptic vesicles in the anterior synapse of excitatory synapse. In contrast, the number of synaptic vesicles in inhibitory synapse is less. In our results, synaptic vesicles in the CUMS-induced model rats were significantly lower than those in the other groups, indicated that excitatory synapses in the CUMS-induced model rats were decreased. On the other hand, widened synaptic cleft increased the distance between presynaptic membrane and postsynaptic membrane and then deferred delivery of the transmitter. All of the changes belonged to synaptic plasticity. When these changes of synaptic plasticity accumulated to a certain extent, the depressive behaviors occurred after MCAO.

There are two main shortcomings in our study. First, the total proportion of newborn astrocytes and newborn neurons was less than 100%, and we did not verify that the rest newborn cells were oligodendroglias or microglias. Second, we did not carefully quantity the changes of GS, SNAT1/2/6/7, GLS, GDH, GABA, and *α*-KG [13] to more clearly explain the details of glutamate circulation. These questions need to be investigated in the future study.

## 5. Conclusions

Our research demonstrated that downregulated astroglial GLT-1 inhibited synaptic plasticity in hippocampal DG of the CUMS-induced model rats by reducing glutamate metabolism. The changes of synaptic plasticity in hippocampal DG of the CUMS-induced model rats promoted the occurrence of depressive behaviors after MCAO. This may indicate one potential pathogenesis of poststroke depression.

## Figures and Tables

**Table 1:** Groups of drug administration.

Group	BrdU	Cef	0.9% saline
Control	+		+
MCAO	+		+
MCAO+CUMS	+		+
MCAO+CUMS+Cef	+	+	

## Data Availability

Raw data were generated at the real-time fluorescent quantitative PCR instrument (StepOne™ Software 2.3, Thermo Scientific, USA), a laser confocal microscope (Olympus, FV1000, Japan), a transmission electron microscope (Hitachi H-7650, Japan), a mass spectrometer (AB Sciex Qtrap 4500, Canada), and so on. Derived data supporting the findings of this study are available from the corresponding author upon request.

## Abbreviations

GLT-1: Astroglial glutamate transporter-1
Cef: Ceftriaxone
EAAT-2: Excitatory amino acid transporter-2
DG: Dentate gyrus
MCAO: Middle cerebral artery occlusion
CUMS: Chronic unpredictable mild stress
SD: Sprague-Dawley
BrdU: 5-Bromo-2′-deoxyuridine
OFT: Open-field test
Sac: Sacrifice
SP: Sucrose preference
PSD: Postsynaptic density
CSF: Cerebrospinal fluid
MS: Mass spectrometry
MRM: Multiple reaction monitoring
ANOVA: Analysis of variance
SEM: Standard error of the mean
SYN, SYP: Synaptophysin
GS: Glutamine synthetase
SNAT: Sodium-coupled neutral amino acid transporters
GLS: Glutaminase
GDH: Glutamate dehydrogenase
*α*-KG: *α*-Ketone glutaric acid.
